# The lived experiences of young people living with type 1 diabetes: A hermeneutic study

**DOI:** 10.1002/nop2.995

**Published:** 2021-07-22

**Authors:** Malin Rising Holmström, Siv Söderberg

**Affiliations:** ^1^ Department of Nursing Mid Sweden University Sundsvall Sweden; ^2^ Department of Nursing Science Mid Sweden University Sundsvall Sweden

**Keywords:** Gadamer, hermeneutic, interviews, nursing, parents, school, transformed and re‐organized everyday life, type 1 diabetes, young people

## Abstract

**Aims:**

The aim of this hermeneutic study was to explore and elucidate the lived experiences of young people living with type 1 diabetes in terms of their everyday life and school in Sweden.

**Design:**

A qualitative interview study with a hermeneutic approach inspired by Gadamer's thinking.

**Methods:**

Interviews were conducted with a purposive sample of seven girls and three boys with type 1 diabetes between January and September 2017 and analysed with a hermeneutic method.

**Results:**

Young peoples' everyday lives were transformed and re‐organized by their illness and they parodically live a double‐edged everyday life. To support young people's healthcare personnel, headmasters and teachers must understand this double‐edged situation.

## 1 INTRODUCTION

This study is part of a larger research project investigating young people's experiences of living with type 1 diabetes (T1D). This hermeneutic study focused on young people's lived experiences at home and in school. T1D is one of the most common endocrine conditions among children globally (Krzewska & Ben‐Skowronek, 2016). In terms of incidence per 100,000 population per year, Sweden (43.2) has the world's second‐highest incidence rate in children (aged 0–14 years) after Finland (62.3) (International Diabetes Federation, 2019). This incidence continues to increase without any indication of a plateau (Ludvigsson, 2017). Being hit by an illness such as T1D leads to a major alteration in everyday life for young people. In Sweden, approximately 700–800 young people develop T1D each year (Swedish National Diabetes Register, 2019); therefore, there are growing numbers of young people in schools with complex health needs as well as needs for individual adjustments and support with self‐care. Educating school staff and the community about the experiences of living with T1D can support young people with the disease in getting help, which is in line with their needs and resources.

### 1.1 Background

Living with a long‐term illness such as T1D can have a significant impact on every aspect of a young people's life both at home and in school. Furthermore, T1D requires knowledge and skills to provide self‐care (Patterson et al., 2014). The standard‐of‐care and self‐care in schools calls for good metabolic control, that is monitoring blood glucose. Good metabolic control requires knowledge of the target glucose numbers and knowledge of self‐care regarding administering insulin by continuous subcutaneous insulin infusion (insulin pumps) or multiple daily insulin injections and continuous glucose monitors including of how to handle a hyperglycaemic or hypoglycaemic emergency (American Association of Diabetes Educators, 2018). In the last decade, there has been a technological explosion in diabetes management devices with the goal of improving quality of life (QOL) (Lee, Im, & Magbual, 2004). Young people spend a great deal of time in school settings, which means that their self‐care is largely performed in schools (Amillategui et al., 2009).

Managing T1D requires young people to be in constant control of their diabetes devices and can create difficulties in interactions with friends and engaging in social life as they did before the onset of T1D (Rising Holmström et al., 2017). Optimal self‐care is crucial for young people's immediate health and well‐being, academic performance and future health (Marks, Wilson, & Crisp, 2013). Living with T1D is a challenge for young people and often requires requiring support from others such as parents, family, friends and healthcare and school professionals, as well as individual adjustments to manage everyday life and school (Hill et al., 2007; Rising Holmström et al., 2017; Rising Holmström et al., 2018). Even though living with T1D can be a challenge for young people, it is important for them to be seen as persons like everyone else—they want to be seen as young people like their peers (Lindberg & Söderberg, 2015).

An earlier study showed that school personnel experienced caring for young people with T1D as challenging with a need for more diabetes competencies (Holmström et al., 2018). Adolescence is a time of life characterized by broad changes, that is physically, mentally and socially, along with a striving for independence and developing a personal identity. In striving for autonomy, young people often need distance from others while still retaining their support (Doherty & Dovey‐Pearce, 2005). This can be especially limiting for young people with a chronic illness such as T1D, which influences this process (Rising Holmström et al., 2017, 2018). Previous research showed that young people with T1D have struggled for normality and independence and needed to make peace with their diabetes to handle everyday self‐management in school, which can be demanding (Rising Holmström et al., 2017; Tuohy et al., 2019). A qualitative phenomenological study showed that the process by which young people navigate living with T1D was characterized by adapting to the diagnosis, learning to live with T1D and becoming independent. Experiential learning was key to adolescents developing self‐management skills and independence (Spencer et al., 2013). A systematic review investigated the prevalence of diabetes distress (DD) finding that about one‐third of adolescents with T1D experience elevated DD, which was frequently associated with suboptimal glycaemic control, low self‐efficacy and reduced self‐care (Hagger et al., 2016).

Type 1 diabetes is a complex illness that influences nearly every aspect of everyday life of the person who has it. This study aimed to elucidate the lived experiences of such people in everyday life and at school. The knowledge can increase the possibility of support for young people with T1D. It is important to remember that these are young people first, and therefore, disease management should concentrate on investing in the future (cf. McDonagh & Gleeson, 2011). Thus, understanding the challenges of living with T1D might allow healthcare and school professionals to recognize the unique needs and concerns of each young person and thereby provide the necessary support.

## 2 THE STUDY

### 2.1 Aims

The aim of this study was to explore and elucidate the lived experiences of young people living with T1D in everyday life and school in Sweden.

### 2.2 Design

We chose a qualitative research design to gain a deeper understanding of young people's lived experiences with T1D. Following this, a Gadamerian‐based research method according to Fleming et al. (2003) was used. The method is based on Gadamer's philosophical hermeneutics, which focuses on interpretation and understanding. According to Gadamer (1994), understanding starts with a question that opens different horizons of understanding. The interrelationship between understanding and questioning is the process of hermeneutic research (Fleming et al., 2003). Here, we found that the aim of the study was consistent with the chosen method.

### 2.3 Participants

A purposive sample of seven girls and three boys with T1D participated in the study based on a model of sample size in qualitative selection and information power according to Malterud et al. (2016). Characteristic variation was attempted by selecting young people from four municipalities who varied in terms of age, gender, family structure, prior diabetic experience, length of time since diabetic onset, use of insulin pump or pen, diagnosis of gluten and ethnicity. The criteria for participation were age over 13 years and diagnosis of T1D for at least one year. The participants were recruited from schools in a county in northern Sweden. School nurses sent letters to parents of students with T1D who met the inclusion criteria. The letters contained information about the study, a request to participate with a response envelope and information about consent to the study. After receiving response letters with consent from parents and young people, the first author contacted the students and arranged for individual interviews at a preferred location of the participant. None of the authors had a prior relationship with any of the participants.

### 2.4 Data collection

Data were collected through in‐depth, open‐ended qualitative interviews with the participants (cf. Kvale & Brinkmann, 2009) between January and September 2017. The interviews were conducted in Swedish and analysed in English. The young people were asked to narrate their experiences of living with T1D. When needed, clarifying questions were asked, such as “Can you give an example?” and “What were you thinking then?” The authors had no earlier relationships with any of the participants. The interviews occurred according to the young people's wishes either in school (*N* = 3), at the library (*N* = 4) or in a quiet room at the university (*N* = 3). The interviews were conducted by the first author, lasted from 3,773 (md = 52) minutes, were recorded and later transcribed verbatim.

### 2.5 Ethical considerations

The study was approved by the Regional Ethics Review Board (Dnr. 2015/416‐31Ö). All participants were informed about the nature of the study and were guaranteed confidentiality and an anonymous presentation of the findings. Informed consent was obtained from parents for participants under 18 years. The participants were informed that their participation was voluntary and that they could withdraw at any time without giving any explanation. This study was conducted in accordance with the Declaration of Helsinki, which supports respect for all human beings and protects their health and rights when participating in research (World Medical Association, 2018).

### 2.6 Data analysis

A hermeneutic approach inspired by Gadamer's philosophy (1994) was used to analyse the interview texts. Fleming et al. (2003) delineated a five‐step research process based on Gadamer's thinking (Table 1). The first step of the hermeneutic analysis started with defining the research question, (i.e. the aim of the study) followed by a reading of the interview texts numerous times to obtain a sense of the whole. During this reading (the second step of the analysis), the aim of the study and the researcher's pre‐understanding are critical. The researchers in this study are registered nurses and researchers in the area of living with long‐term illness. The third step consists of a dialogue with the participants, that is interviews. The fourth step involved a dialogue with the interview text whereby units of meaning were identified, condensed, related to each other and grouped into six subthemes and one theme based on similarities and differences in meaning.

**TABLE 1:** Steps of data analysis according to Fleming et al. (2003)

Step 1	Deciding upon a research question
Step 2	Identifying the researcher's pre‐understanding which is according to Gadamer (1994) the only way to reach an understanding. Without recognizing pre‐understanding, there is a risk that meaning will be misunderstood or misjudged
Step 3	Gaining understanding through dialogue with participants, in this study in form of interviews with young persons with T1DM
Step 4	Gaining understanding through dialogue with the interview text, which means identifying units of meaning that corresponds to the aim of the study
Step 5	Establishing trustworthiness (Rigour)

### 2.7 Trustworthiness‐Rigour

The last and the fifth step in the data analysis process according to Fleming et al. (2003) is to establish trustworthiness. The trustworthiness of this study agreed with Fleming et al. (2003) who found that the responsibility of a Gadamerian researcher is to give sufficient information on the research process. Here, credibility, confirmability and truthfulness are aspects of trustworthiness that we considered. Direct quotations from the interviews increase readers’ ability to judge the credibility of the study. Confirmability can be established by returning to the participants during the research process. To meet this criterion, we returned to the interview texts throughout the research process as we found this to be sufficient; therefore, we did not return to the participants. Concerning truthfulness, Gadamer (1994) meant that there is no one universal truth. This means that understanding can be established only when agreement among the parts and the whole is reached. In this study, we were aware of our pre‐understanding as registered nurses and nursing researchers in the area of living with long‐term illness—this experience affects our understanding and interpretation during the research process. Our aim was not to present a single truth, but rather to present the most probable interpretation based on our pre‐understanding and the textual analysis.

## 3 FINDINGS

The study participants ranged in age from 13–18 years (md = 15 years) with a length from onset ranging from 414 years (md = 10 years). The participant's details are presented in Table 2.

**TABLE 2:** Sample characteristics

Name and gender Female (F) Male (M)		Age	Length of time diagnosed with T1D	Place of birth Sweden (S) Europe (E) Municipality (1–4)	Two‐parent‐family (2PF)	Diagnosed with gluten intolerance (GI)	Prior diabetic experience from relatives	Insulin pump (PU) Insulin pen (PE)
1	Amanda F	18	12	S 1	2PF		T2D	PU
2	Beatrice F	14	10	E 1	2PF			PU
3	Cecilia F	17	10	S 1	2PF			PU
4	Debbie F	16	11	S 4				PU
5	Eric M	14	10	E 2	2PF	GI		PE
6	Frank M	14	6	S 3	2PF		T2D	PE
7	Gloria F	18	14	S 3	2PF			PU
8	Hanna F	15	4	S 4			T1D	PE
9	Isabell F	15	5	E 2	2PF		T2D	PU
10	Jacob M	13	9	S 2	2PF	GI		PU

Note

Median for age = 15 years.

Median for length of time diagnosed with T1D = 10 years.

Findings disclosed the multifaceted everyday life that the young people with T1D faced. One overall theme, *Living a transformed and re‐organized everyday life*, was formulated to describe how young people experienced the changes and impact of everyday life they encountered with the onset and development of their illness.

### 3.1 Living a transformed and re‐organized everyday life

This theme was formulated from the following six subthemes: feeling new emotions in the body, living a governed everyday life, being affected as a person, being met with understanding and support, informing about diabetes is important and school can be problematic. The subthemes are explained below and are illustrated with verbatim quotations from the interviews.

#### 3.1.1 Feeling new emotions in the body

The young people with T1D experienced the first sign of diabetes as something felt wrong in their body—something they did not recognize and had experienced earlier in life. This “something” was the beginning of the process of being diagnosed with T1D. They experienced a novel feeling of being sick, and they could not understand why they had this feeling. Young people were very thirsty with a new kind of thirst—a thirst that was constantly present. They consequently had to urinate frequently. Some were affected by infections and/or lost weight even though they ate a lot, were always hungry and lacked energy.

At first, I was very hungry and ate like a horse, and at the same time I lost weight and had to pee all the time…and I was thirsty, a new kind of thirst I never felt before.

Because of signs that something was not as it should be, contact was sought with the school nurse, the healthcare centre or the hospital by one of their parents—often their mother. After being tested with different assessments, T1D was identified as the cause. After that, the diagnosis of T1D was determined and they were remitted to a nearby hospital for further investigation, treatment and learning to manage the treatment necessary for the illness. This was often the first time that the young people had met healthcare providers as a patient.

In the beginning, everything was just awful…. I refused everything and I wondered why this has happened to me?! WHY ME!

#### 3.1.2 Living a governed everyday life

Young people with T1D described that their everyday life was ruled by T1D. They expressed that living with T1D meant living with a troublesome illness that was never going to disappear. Young people emphasized that T1D is an individual illness, and it was important to know their illness and to have control. They wanted to learn to manage their illness, but at the same time, it was difficult to know how they wanted to manage it—for example whether to use the insulin pump or the insulin pen. Young people described that the whole family was impacted, and they experienced that their parents were worried and burdened and had expressed grief and sadness when the diagnosis was verified. It was important for them to participate in the management of their illness because their parents took responsibility for the treatment especially at the onset of T1D. First and foremost, it was the mother who often administered insulin. She contacted the school, teachers and headmaster.

At first, Mum was in school with me and then she came to school for lunch and helped me with the insulin. Then Mum taught the teachers how to help me manage with blood sugar and all.

Young people had to learn to use different kinds of technical aids, for example continuous blood glucose meters and insulin pumps. They used these devices so that they had more freedom, which had a positive impact on their everyday life.

I've got a wireless CGM (continuous glucose meter) that I manage myself as well as a pump—it's so good, so much more freedom.

Young people were very aware of the importance of food for people who have diabetes, and they had to learn how to handle food and meals related to T1D. They also had to learn to count carbohydrates, which was occasionally hard to manage, and they discovered that there is a difference between fast and slow carbohydrates.

I've been to the dietician and learned how to count carbs; I could not cope. I can't be like a calculator the rest of my life.

#### 3.1.3 Being affected as a person

Young people with T1D described that it was a huge readjustment to be diagnosed with T1D. They knew the date for the onset of T1D, and they did not celebrate this occasion but remembered because it symbolized an irreversible change in their everyday life. Suddenly they were seriously ill with a disease that they did not know very much about. After diagnoses, they experienced being seen as a different person by people in their environment. In school, they might experience that they were seen as a disease and that friends thought diabetes was disgusting or strange.

It felt like I was regarded as a completely different person in school almost like as I was a disease….

Young people described how it affected and frightened them when they saw how worried and sad their parents were over the symptoms of T1D; for example, their mother cried over the symptoms of T1D, and they did not understand why. Also, feelings of sadness arose when it was clear that it was diabetes that caused the ill‐health they had experienced and there was no cure. One young person expressed:

I remember Dad's hands trembling when he was going to check my blood glucose for the first time…. I suddenly realized he was nervous and scared…and that made me scared too.

It was important for young people to manage most of the details related to their illness by themselves. They wanted to have control over the situation and their body and expressed that they practised how it felt to be low or high in blood glucose so that they could recognize the feeling. This helped them to be aware of symptoms and to gain control but having to always be on their toes caused a lot of stress. They described that their mothers had a significant role and were a great support when it came to managing their T1D, even though they tried to ask for help only when they did not have the energy to manage it by themselves, for example to change needles.

I only ask Mum to help me switch needles when I’m tired.

#### 3.1.4 Being met with understanding and support

Young people with T1D described that having support was essential for them to manage to live with their illness. Their parents, especially the mothers, have a central role in giving support. Young people with T1D described using their cellphones to get continuous support from their parents regarding self‐management.

Mum and Dad gave me a mobile and I called them from school several times a day and said, “my blood sugar is so and so I have this on my plate,” and then they said how much insulin I should take…. They are constantly on standby for me, even today.

Young people described the school environment as stressful and reported that this affected their blood glucose level. The stress could be caused by many different things common to all young people such as homework and examinations and being a young person trying to fit in. It can also be related to diabetes such as always being on alert for symptoms so they could have control of the illness. The young people described not feeling secure in school because they were afraid that nobody could help them if they had hypoglycaemia or hyperglycaemia.

I sometimes neglect to check the blood sugar in school…because it is a bit awkward to go away from class and test…. It takes time…. I miss class…so the blood sugar is not so accurate at school…often a bit high…. I can get stressed over this… There is so much I have to do…then I get nervous…and then my blood sugar usually rises…and then afterwards it turns low, and I get a hypo…and then I get all shaky and trembling.

I do not feel safe at school…. I’m not sure people would notice if I got a hypo….

Young people with T1D had continuous contact with the diabetes nurse and physician at the hospital, but they did not highlight them as the most important support. Another way of receiving support was to be able to openly take insulin or show their insulin pump especially with their friends around. Young people expressed that having friends who were nice and helpful was critical. They described different situations when they had experienced being met with understanding, for example when they were reminded by friends that their blood glucose may be low and that they should eat something. Another example was when they could speak freely and tell the class and teacher that their blood glucose was low, and they had to leave the classroom to get something to eat.

Now I manage my T1D completely openly and if someone asks do you have diabetes? I just say, Yes, I do!

The participants expressed that they could receive understanding and support from teachers in school. They appreciated teachers who keep dextrose on hand for them and those who show understanding when they have to leave lessons because they are not feeling well.

#### 3.1.5 Informing about diabetes is important

Young people with T1D emphasized that it was very important to spread information about T1D to everyone to counteract prejudices. They experienced that there is a lack of knowledge in society about diabetes. For example, young people underlined that many people did not know that there are two types of diabetes and that they are caused by different things. They had experienced that some people thought that T1D was a disease caused by eating too much sugar likely because of newspaper reports that diabetes is increasing due to people eating too much sugar and having an unhealthy lifestyle without clarifying that this is in reference to type 2 diabetes (T2D).

More information to all so I don't have to hear that it's my own fault because I’ve eaten too much sugar.

It was essential and necessary to inform various people in school in addition to teachers and headmasters, for example the staff responsible for cooking and the school nurses. Young people with T1D described getting blamed for and accused of causing the illness themselves through an unhealthy lifestyle. This was very frustrating and hurtful for the young people who struggled to manage everyday life.

I've been told so many times by adults, people I don't know at all, it's your own fault you got diabetes; you eat a lot of sweets…. At first, I just got sad, now I get angry…. And it depends on who is saying it…. If it's someone I know who does not know…then I usually explain and say that I’ve not done anything wrong and Type 1 diabetes is not caused by eating sweets.

#### 3.1.6 School can be problematic

Young people with T1D described that school was a problematic place to be for a student with T1D. They met teachers and headmasters who did not care that they had a long‐term illness. Young people expressed that all teachers needed knowledge and education about T1D. Their parents had to contact the school and inform staff about T1D because young experienced that no one else was responsible for that task. Parents (and often the mother) had to inform and educate teachers about what it means to have a diagnosis of T1D and what follows the diagnosis. Young people expressed that some teachers and headmasters showed no understanding about their illness.

It was strange that no one cared… it was like no one was responsible and no one cared and the headmaster thought it was so much fuss with me and told Mum not to enlarge problems.

The school lacked knowledge about diet and students with T1D and an understanding that T1D demanded a set schedule for meals and that access to snacks is an absolute necessity.

The ladies in the kitchen wanted the best for me…but it got hard when they kind of chased me with the plate. Mom talked to the school and explained what a normal serving was for me and eventually it got better.

## 4 DISCUSSION

This study sought to investigate young people's lived experience of living with T1D. The participants’ lives were greatly influenced by their illness (i.e. the theme Living a transformed and re‐organized everyday life). The findings were interpreted in the light of Toombs's (1993) work on understanding the meaning of illness for sick persons and prior research. The journey to being diagnosed with T1D started for the young people with an experience of new emotions in the body including feelings that they had not felt before. This means that their entire life situation was altered. This can be seen as what Toombs (1993) called a loss of the familiar world. When a person is healthy, everyday life is largely taken for granted, but being stricken by illness destroys this taken‐for‐grantedness (Toombs, 1993).

The illness interrupted the young people's involvement in the world, and the lived experience of the body became the centre of attention. Young people with T1D described their life as ruled by their illness, and they understood that T1D would never disappear. Bury (1982) described chronic illness such as T1D as a disruptive event—a disruption of what is taken for granted in everyday life. For young people, a T1D diagnosis means that they must learn to live with an illness that is ever‐present in everyday life and will never disappear. According to Toombs (1993), such illness became a fundamental loss of wholeness that manifested in several forms. Further, Toombs noted that it is important to recognize that loss of wholeness is also an acute awareness of loss of control of the present situation, and the freedom to act is conceded. For young people with T1D, this means that they live in what Kelleher (1988) called a double‐edged condition. The condition demands adherence to medical treatment and control of their blood glucose for staying well. At the same time, this means a loss of freedom and control over their life.

Young people with T1D described that the onset of the illness affected them as persons undergoing a huge transformation from being young and healthy to being a person with T1D. The illness suddenly took over their bodies, and the young people did not recognize the reactions from their own bodies. Furthermore, they had little knowledge about T1D and had to learn how to manage everyday life in a new way. This could be compared to a disruption of the lived body as described by Toombs (1993). Illness means disability—the inability to engage in the world in habitual ways. Furthermore, young people with T1D experienced no longer being seen as the same person as before by people around them but rather were seen as the illness itself. Research has shown that a challenge for young people with T1D was feeling different (DeCosta et al., 2020) from friends and being afraid of losing friends and being alone (Freeborn et al.., 2013). The young people with T1D had to reorientate to a new way of life with T1D.

Being met with understanding and support from parents and friends was of utmost importance for the participants and helped them to manage their illness in a positive way, which is in line with empathic understanding as described by Toombs (1993). Research has shown that parents are important for supporting their child in the managing of diabetes (Akre & Suris, 2014; DeCosta et al., 2020). Supportive parents are related to high metabolic control (Bowen, Henske, & Potter 2010; Scholes et al., 2013). The participants emphasized that it was first and foremost their mothers who provided support in handling the illness. According to Lindberg and Söderberg (2015), the parents, but especially the mother, gave the most important support. An interesting finding is that the participants did not call attention to the support and care they received from healthcare personnel. This may reflect that diabetes care in Sweden focuses on a transition of responsibility and management of the illness to the young person and their parents. The young people with T1D experienced a lack of knowledge about T1D in their communities and emphasized that informing the public about diabetes was most important for preventing prejudice. They especially noted that it was important to spread knowledge about the differences between T1D and T2D. Studies have shown that young people with T1D kept their illness secret because of this prejudice (Seo et al., 2020) and found it difficult to talk about diabetes with their peers as they experienced situations of rejection and shame (Sparapani et al., 2015).

Parents, especially mothers, informed and trained teachers about diabetes to improve the school situation for their children. Even so, school can be problematic for young people with T1D, and the findings show differences in perspectives of understanding and lack of knowledge of the impact of T1D. The school staff needs to gain a more complete understanding of the young people's lived experience of T1D to meet the young people in the *healing relationship* described by Toombs (1993). Earlier research showed that children with T1D need supportive relationships (DeCosta et al., 2020). There is indeed a need for relationships based on a shared understanding of the meaning of illness for an adequate understanding of the young people's lived experience of T1D, which is important for ensuring effective support for self‐management in school.

### 4.1 Limitations

The findings of this study cannot be generalized because the intention of qualitative research is not generalization. Rather, these findings can be recontextualized and transferred to similar situations, that is young people living with a long‐term illness (cf. Polit & Beck, 2021). In this study, the participants were recruited following a purposive sampling procedure. A criticism of purposive sampling is that the method encourages a certain type of informants (cf. Morse, 1991). However, this critique does not consider that purposive sampling is used as a tool to provide a theoretical richness in attempting to describe the phenomenon as fully and accurately as possible. The number of participants was considered to be sufficient to reach variation and to sustain depth in the analysis. In reducing the risk of biased decisions and idiosyncratic interpretations, the researchers used investigators’ triangulation (cf. Polit & Beck, 2021). This means that the researchers coded the interview texts separately and then compared and discussed the coding until a consensus on subthemes and themes was reached.

## 5 CONCLUSION

This study shows that for young people with T1D, everyday life at home and in school are governed and transformed by their illness. This means that they paradoxically live in a double‐edged condition and situation: the illness demands adherence to treatment and control of blood glucose levels for staying well but achieving this can generate a feeling of loss of freedom and decreased autonomy in everyday life (cf. Kelleher, 1988). Parallel to this doubled‐edge situation, young people with T1D are first and foremost young people. They face the same challenges as their peers in striving for independence from parents and others by taking responsibility for themselves as they move towards adulthood. To support young people with T1D, healthcare personnel, headmasters, and teachers must understand this complex situation that governs young people's everyday life. Knowledge and understanding of this situation implies that young people can receive support tailored to their needs thus decreasing their feelings of being governed by the T1D.

## CONFLICT OF INTEREST

No conflict of interest has been declared by the authors.

## AUTHOR CONTRIBUTIONS

All authors meet at least one of the criterias recommended by the ICMJE: (http://www.icmje.org/ethical_1author.html) and have agreed on the final version.
